# HbA1c Test as a Tool in the Diagnosis of Gestational Diabetes Mellitus

**DOI:** 10.1371/journal.pone.0135989

**Published:** 2015-08-20

**Authors:** Paula Breitenbach Renz, Gabriela Cavagnolli, Letícia Schwerz Weinert, Sandra Pinho Silveiro, Joíza Lins Camargo

**Affiliations:** 1 Graduate Program in Endocrinology, Universidade Federal do Rio Grande do Sul (UFRGS), Porto Alegre, Brazil; 2 School of Medicine, Universidade Católica de Pelotas (UCPEL), Pelotas, Brazil; 3 Endocrinology Department, Hospital de Clinicas de Porto Alegre (HCPA), Porto Alegre, Brazil; Broad Institute of Harvard and MIT, UNITED STATES

## Abstract

**Aims:**

Gestational diabetes mellitus (GDM) is a prevalent and potentially serious condition which may put both mothers and neonates at risk. The current recommendation for diagnosis is the oral glucose tolerance test (OGTT). This study aimed to determine the usefulness of HbA1c test as a diagnostic tool for GDM as compared to the traditional criteria based on the OGTT.

**Methods:**

This was a diagnostic test accuracy study. We performed OGTT and HbA1c test in women attending prenatal visits at a tertiary hospital. GDM was defined according to WHO1999 or ADA/WHO 2013 criteria. ROC curve was used to evaluate the diagnostic performance of HbA1c. Sensitivity, specificity and likelihood ratios for different HbA1c cut-off points were calculated.

**Results:**

Of the 262 women in the third trimester of gestation enrolled in the study, 86 (33%) were diagnosed with GDM. Only five of these women presented HbA1c ≥48 mmol/mol (6.5%). This cut-off point presented 100% specificity but very low sensitivity (7%). Based on ROC curve, and considering OGTT as the reference criterion, HbA1c ≥40 mmol/mol (5.8%) showed adequate specificity in diagnosing GDM (94.9%) but low sensitivity (26.4%). Unlike, HbA1c values of 31 mmol/mol (5.0%) presented adequate sensitivity (89.7%) but low specificity (32.6%) to detect GDM. For women with HbA1c ≥40 mmol/mol (5.8%), the positive and negative likelihood ratios were 5.14 (95%CI 2.49–10.63) and 0.78 (0.68–0.88), respectively. The post-test probability of GDM was about 40%, representing a 4.0-fold increase in the mean pre-test probability. This cut-off point could eliminate the need for the unpleasant and laborious OGTT tests in almost one third of cases, as 38% of patients with GDM may be diagnosable by HbA1c test alone.

**Conclusions:**

Our results show that combined HbA1c and OGTT measurements may be useful in diagnosing GDM.

## Introduction

Gestational diabetes mellitus (GDM) is a prevalent and potentially serious condition that may lead to adverse effects in both mothers and neonates [1]. It is associated with preeclampsia, increased caesarean rates, and macrosomia [2, 3]. The detection and treatment of this condition reduce the risks for the mothers as well as for the babies [4,5].

Although the risks of complications in the presence of GDM are well established, there is considerable controversy regarding its diagnosis [6]. Traditionally, the OGTT has been the test of choice for this condition. It can be preceded by a screening strategy such as fasting glycemia (FG) or a glucose load test. However, there are still divergences as to the OGTT cut-offs which should be used for the diagnosis of GDM and also a recent review concluded that the evidence are insufficient to permit assessment of which strategy is best to diagnose GDM [7, 8].

In 2010, the American Diabetes Association (ADA) included HbA1c test as a diagnostic criterion for diabetes (DM) in the general population. The cut-off of HbA1c ≥48 mmol/mol (6.5%) was established for the diagnosis, and was endorsed by the World Health Organization (WHO) in 2011 [9, 10]. This cut-off has high specificity in diagnosing DM [11,12,13]. However, HbA1c and glucose tests show very weak agreement, and it seems that these two tests may identify different populations of patients [11].

Recent results from the Hyperglycaemia and Adverse Pregnancy Outcomes (HAPO) study showed that HbA1c measurements, similar to glycemia levels, were significantly associated with all adverse outcomes, and higher levels of maternal HbA1c were related to a greater frequency of adverse outcomes [14].

Although OGTT is accepted as the diagnostic test for GDM by international organizations, it requires at least 8h fasting, extensive patient preparation, lacks reproducibility, it is time-consuming and unpalatable. Unlike, HbA1c may be measured any time of the day, has less biological variation, higher reproducibility and better analytical stability as compared to glucose measurements [15]. Additionally, HbA1c test does not need fasting and would be more comfortable for pregnant women than the OGTT. Nevertheless, its use for the diagnosis of GDM has not yet been recommended by any current guidelines.

The aim of this study was to analyse the performance of the HbA1c test in detecting GDM, based on OGTT as the reference test.

## Patients and Methods

**Fig 1:**
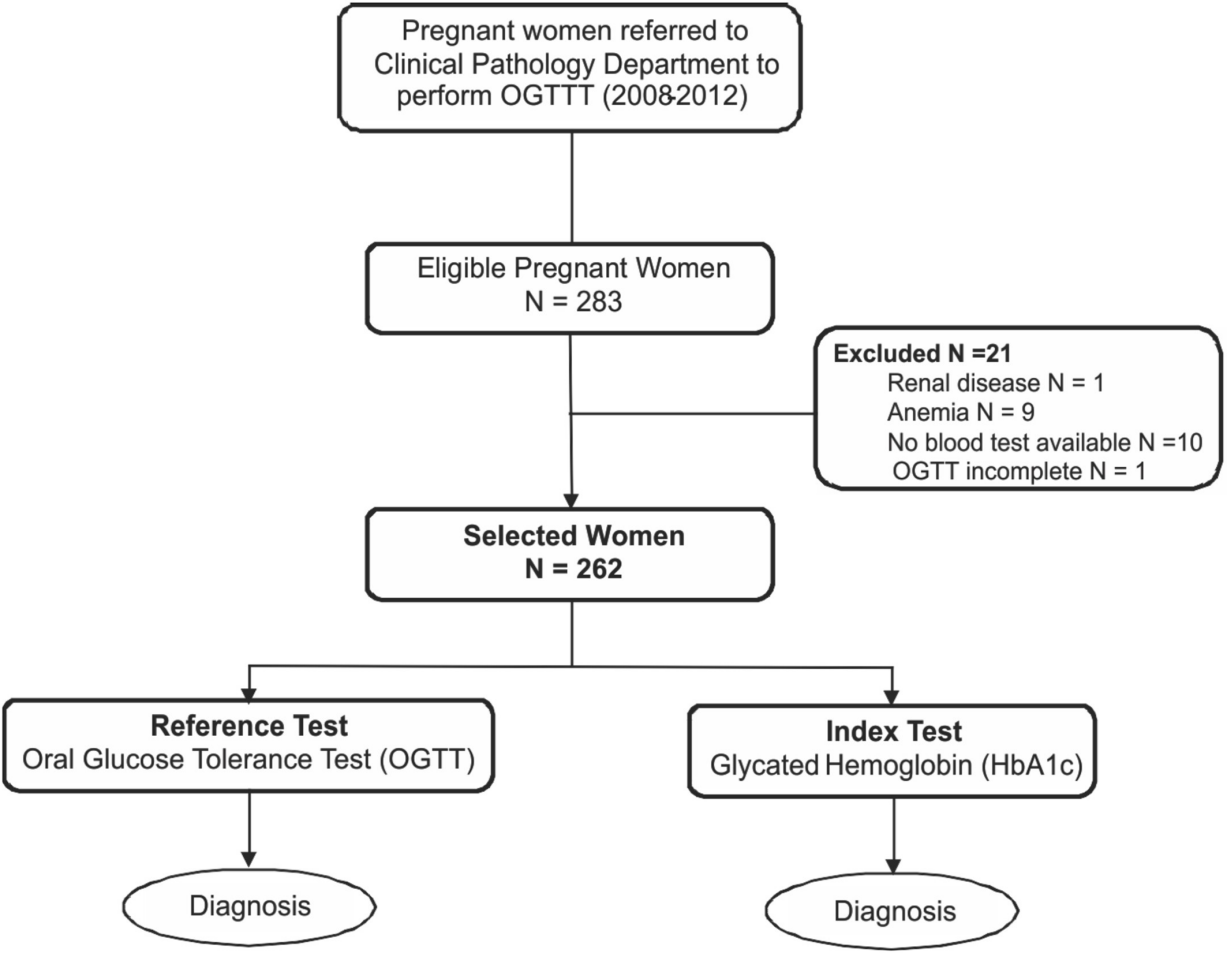
Study Design.

This was a study of diagnostic test accuracy to evaluate the performance of the HbA1c test in diagnosing GDM. Our findings were presented according to Standards for Reporting of Diagnostic Accuracy (STARD) initiativ*e* guidelines [16] (**Fig 1**).

Pregnant women in prenatal care, without previous DM, who were referred to the Clinical Pathology Department of the Hospital de Clinicas de Porto Alegre (HCPA) between September 2009 and July 2012 to perform OGTT tests in the third trimester of pregnancy were consecutively invited to participate in the present study. All women signed an informed consent form and answered a standardized questionnaire. Age, gestational age, obstetric history, smoking, family history of cardiovascular disease (CVD), DM, arterial hypertension (HT), alcohol consumption and medication use were recorded. Patients’ weight and height were also recorded, and used to calculate BMI (kg/m^2^) values.

Patients with the following conditions, which are known to interfere with or lead to the misinterpretation of HbA1c results, were excluded from participation: anemia, chronic renal disease and/or presence of hemoglobin variants [17].

After an overnight fast, blood samples were drawn to determine HbA1c levels, blood cell counts, lipid profile, creatinine and glucose concentrations. The OGTT was performed according to current recommendations [1,9]. Plasma glucose and serum lipid levels were determined by enzymatic methods, and creatinine concentrations were estimated by the Jaffe reaction (Modular P, Roche Diagnostics, Basel, Switzerland). Hemograms were performed by flow cytometry (ABX Pentra DX 120, HORIBA, Kyoto, Japan). HbA1c levels were determined by a HPLC method (Variant II Turbo HbA1c, BioRad Laboratories, Hercules, CA, USA). This method is a National Glycohemoglobin Standardization Program (NGSP) certified method (http://www.ngsp.org/prog/index.html), and is aligned with International Federation of Clinical Chemistry (IFCC) reference method. The Clinical Pathology department of the HCPA is a participant of the HbA1c External Quality Assurance Program, in which it has shown adequate peformance. GDM was diagnosed according to WHO 1999 criteria (FG ≥7.0 mmol/L and 2h ≥7.7 mmol/L); after June 2011, we also included patients diagnosed through ADA/WHO 2013 criteria (one out of three of the following cut-off points: fasting glycemia ≥5.1 mmol/L, 1 h ≥10.0 mmol/L and 2 h ≥8.5 mmol/L) [1,9,18]. The final diagnosis according to either WHO1999 or ADA/WHO 2013 criteria was considered for data description and summarization purposes.

The present study was approved by the Research Ethics Committee of the Hospital de Clínicas de Porto Alegre (HCPA), under protocol number 10–0475.

### Statistical analyses

Data were expressed as mean and SD for normally distributed variables, and as median (range) for non-Gaussian variables. Student’s *T*-tests, Mann–Whitney *U* tests, McNemar tests and kappa coefficients were used as appropriate. Receiver Operating Characteristic (ROC) curve was used to analyze the performance of the HbA1c test in diagnosing GDM considering the OGTT as reference diagnostic criteria. For this analysis, we initially considered patients diagnosed according to WHO 1999 (N = 262) and ADA/WHO 2013 criteria (N = 145) separately. An analysis was then performed including patients diagnosed according to both criteria. A ROC statistics based upon logistic regression analysis considering the covariates “previous history of GDM”, “age” and “BMI” in the model were also carried out. Sensitivity, specificity and likelihood ratios (LR) for different HbA1c cut-off points were calculated. LR greater than 1 for a positive test is associated with the presence of the disease (LR+), while LR lower than 1 for a negative test is associated with the absence of the disease (LR–) [19]. To increase the clinical applicability of the present results, we also estimated the post-test probability of GDM using the Fagan nomogram [20] considering a pre-test probability of 10%, which corresponds to the mean worldwide prevalence of GDM [21]. A significance level of 5% was adopted for all tests, and the IBM SPSS software for Windows, version 19.0 (*Statistical Package for Social Sciences—Professional Statistics*, IBM Corp., Armonk, USA) was used for all statistical analyses, except for ROC curves comparison where the R-project/pROC was used.

## Results

Of the 283 pregnant women recruited, 21 were excluded (1 renal disease, 9 anemia, 10 no blood sample available and 1 OGTT incomplete) and 262 were included in the study, and were assessed as to the presence or absence of GDM according to WHO 1999 criterion. A total of 145 of these patients were also classified with or without GDM according to ADA/WHO 2013 criteria. All patients were in the third trimester of pregnancy (gestational age = 27±5 weeks) and presented ages between 23 and 35 years. The clinical and laboratory characteristics of study participants are depicted in **Table 1**.

**Table 1:** Clinical and laboratory characteristics of pregnant women with and without GDM, according to WHO 1999 or ADA/WHO 2013^a^ diagnostic criteria.

	WHO 1999 and/or ADA/WHO 2013 OGTT		
	− GDM	+ GDM	
	N = 176	N = 86	*P*
**Age (years)**	28 ± 6.3	32 ± 5.7	<0.001
**Gestational Age (weeks)**	27 ± 5.1	26 ± 5.5	0.152
**BMI (kg/m2)**	29 ± 6.4	32± 5.4	<0.001
**SBP (mmHg)**	111 ± 12.6	117 ± 14.8	<0.001
**DBP (mmHg)**	66 ± 9.6	74 ± 10.5	<0.001
**FG (mmol/L)**	4.5 ± 0.4	5.2 ± 0.9	NA
**1hG (mmol/L)** ^**a**^	6.7 ± 1.4	9.6 ± 1.7	NA
**2hG (mmol/L)**	6.0 ± 1.0	8.8 ± 1.8	NA
**HbA1c [mmol/mol (%)]**	32 ± 4 (5.1 ± 0.4)	37 ± 5 (5.5 ± 0.5)	<0.001
**Hb (g/dL)**	12 ± 0.8	12 ± 0.8	>0.500
**Cholesterol (mg/dL)**	212 (± 40,5)	229 (± 53,5)	0.2510
**HDLCholesterol (mg/dL)**	51 (± 13,3)	59 (± 16,9)	<0.001
**FHDM (%)**	59.5	66.0	0.419
**PHGDM (%)**	4.5	11.8	<0.01
**PHHT (%)**	19.4	20.6	0.832

^a^ After June 2011.

Data are mean ± SD, except for FHDM, PHGDM and PHHT. GDM = gestational diabetes mellitus, OGTT = oral glucose tolerance test, BMI = body mass index, SBP = systolic blood pressure, DBP = diastolic blood pressure, FG = fasting glycemia, 1hG = glycemia after 1h of glucose load, 2hG = glycemia 2h after glucose load, Hb = haemoglobin, FHDM = family history of DM, PHDMG = previous history of GDM, PHHT = previous history of hypertension.

A total of 86 women (33%) presented with GDM, of whom 72 were diagnosed by WHO 1999 criteria. Twenty-six of these women were also diagnosed according to ADA/WHO 2013 criteria, while 14 patients were only diagnosed by ADA/WHO 2013 criteria. The WHO 1999 and ADA/WHO 2013 criteria (N = 142) showed good diagnostic agreement (kappa = 0.639; p <0.001).

There was a statistically significant difference in the age, BMI, blood pressure and history of GDM between women with and without GDM (P <0.001). HbA1c values were 37 ± 5 mmol/mol (5.5 ± 0.5%) and 32 ± 4 mmol/mol (5.1 ± 0.4%) for pregnant women with and without GDM, respectively (P <0.001).

**Fig 2:**
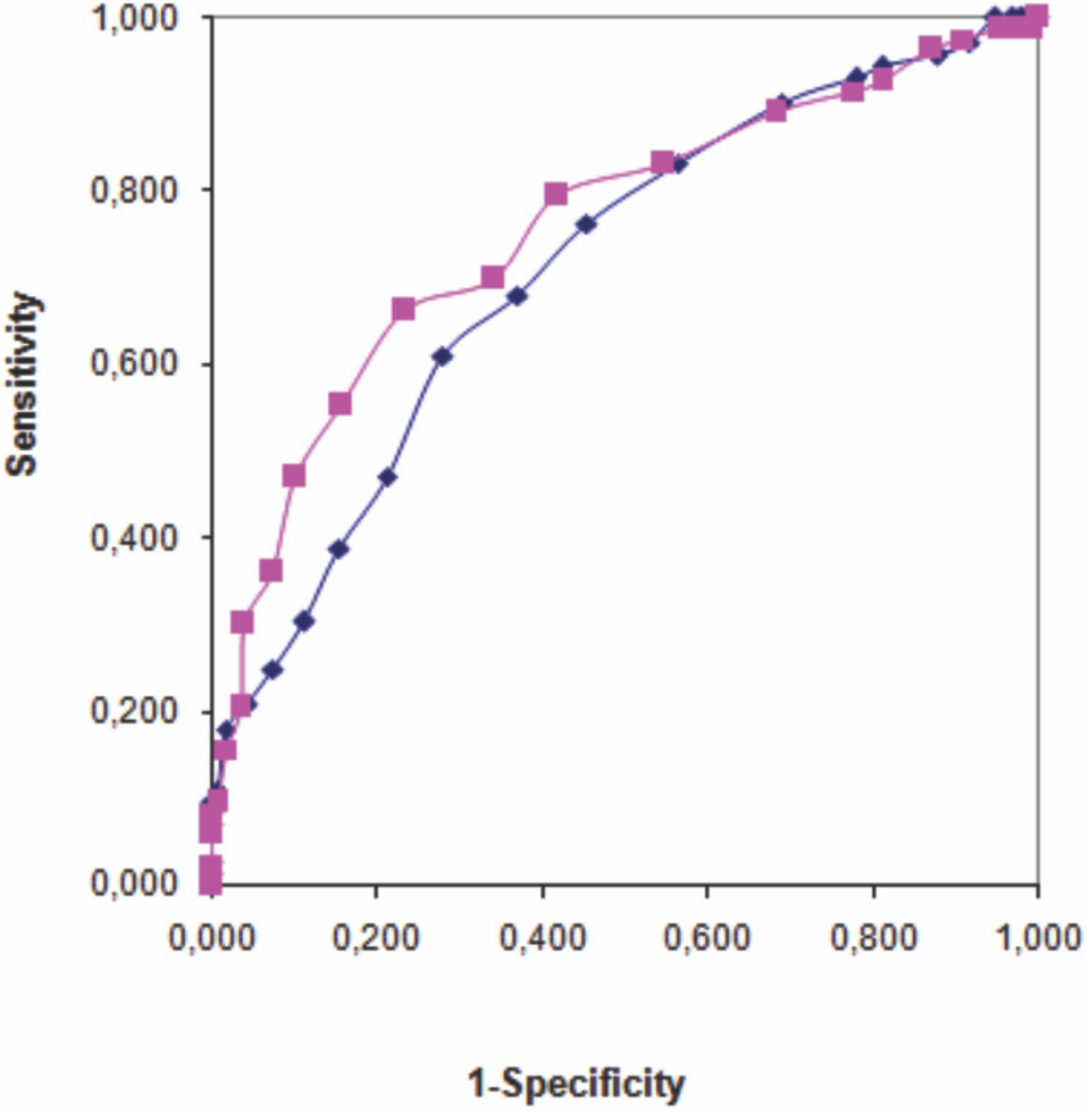
ROC curve for HbA1c values in diagnosing GDM. Blue Line—HbA1c *vs* WHO 1999 N = 262, Red line—HbA1c *vs* ADA/WHO 2013* N = 142. * After June 2011.

A ROC analysis (**Fig 2, Table 2**) considering WHO 1999 criteria alone as the reference test found an AUC of 0.714 (p <0.001) and the cut-off point obtained by the point with the best equilibrium between sensitivity and specificity (100%-to-100% diagonal) was HbA1c value of 35 mmol/mol (5.3%). This cut-off point presented sensitivity and specificity rates of 68.1% and 63.2%, respectively. Similar results were obtained when ADA/WHO 2013 criteria alone were used as reference for the ROC analysis.

**Table 2:** HbA1c test performance in diagnosing GDM.

HbA1c [mmol/mol (%)]	Sensitivity (%)	Specificity (%)	LR+ (95% CI)	LR–(95% CI)
31 (5.0)	89.7	32.6	1.33 (1.17–1.51)	0.32 (0.17–0.61)
32 (5.1)	83.9	44.3	1.56 (1.32–1.84)	0.36 (0.21–0.58)
33 (5.2)	78.2	58.3	1.87 (1.52–2.31)	0.37 (0.25–0.57)
34 (5.3)	70.1	66.9	2.12 (1.65–2.72)	0.45 (0.32–0.63)
36 (5.4)	63.2	76.0	2.63 (1.93–3.59)	0.48 (0.36–0.65)
37 (5.5)	50.6	82.9	2.95 (2.00–4.34)	0.60 (0.48–0.75)
38 (5.6)	41.4	88.0	3.45 (2.15–5.53)	0.67 (0.55–0.80)
39 (5.7)	31.0	90.9	3.39 (1.93–5.96)	0.76 (0.65–0.88)
40 (5.8)	26.4	94.9	5.14 (2.49–10.63)	0.78 (0.68–0.88)
41 (5.9)	20.7	97.1	7.24 (2.78–18.85)	0.82 (0.73–0.91)
42 (6.0)	15.0	98.3	8.72 (2.55–29.79)	0.79 (0.79–0.95)
AUC			0.757	

LR+ = positive likelihood ratio; LR− = negative likelihood ratio. N = 262, WHO 1999 and/or ADA/WHO 2013 reference criteria.

When both WHO 1999 and ADA/WHO 2013 criteria were considered, the AUC was 0.757 (p <0.001) and the cut-off points obtained were very similar to those obtained when each set of criteria were used separately. The ROC statistics based upon logistic regression analysis considering the covariates “previous history of GDM”, “age” and “BMI” in the model showed an AUC slightly different but with no statistical difference (AUC adjusted = 0.787; p >0,05).

**Fig 3:**
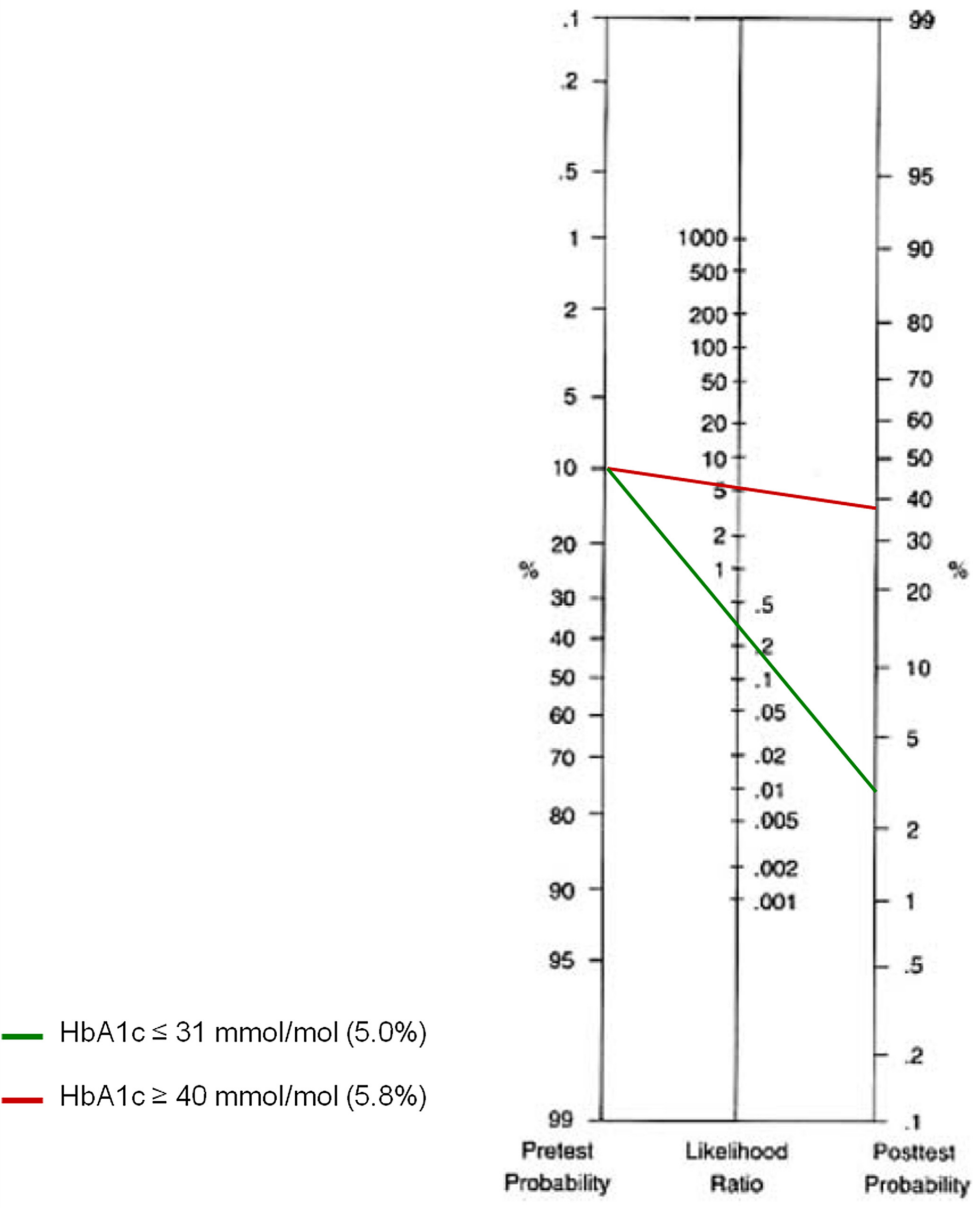
Fagan´s Nomogram for the HbA1c test showing post-test probabilities of gestational diabetes in pregnant women with HbA1c ≤31 mmol/mol (5.0%) and HbA1c ≥40 mmol/mol (5.8%).

The LR+ and LR–were also calculated for the HbA1c values obtained (**Table 2**). HbA1c values of 31 mmol/mol (5.0%) and 40 mmol/mol (5.8%) were the first points in the ROC curve with sensitivity and specificity values over 90%, respectively. HbA1c ≥31 mmol/mol (5.0%) had a LR+ of 1.33 (95% CI 1.17 to 1.51) and a LR–of 0.32 (95% CI 0.17 to 0.61); conversely, HbA1c ≥40 mmol/mol (5.8%) exhibited a LR+ of 5.14 (95% CI 2.49 to 10.63) and a LR–of 0.78 (95% CI 0.68 to 0.88). Considering a pre-test probability of 10% for GDM, the post-test probabilities for GDM were approximately 3.5% and 40% for HbA1c ≤31 mmol/mol (5.0%) and HbA1c ≥40 mmol/mol (5.8%), respectively (**Fig 3**).

Based on these data, and considering HbA1c ≥40 mmol/mol (5.8%) as rule in cut-off point for GDM, 32 pregnant women were classified with GDM. These women were more likely to report previous GDM and a family history of DM, tended to be older, and to have higher BMI values, blood pressure (systolic and diastolic), glycemia (fasting, 1h and 2hG) and cholesterol levels. Five of these women presented with HbA1c ≥48 mmol/mol (6.5%).

The agreement between the diagnoses provided according to the HbA1c cut-off adopted in this study and the OGTT results was fair (kappa = 0.253; p <0.001). Of the 72 women classified as having GDM according to WHO 1999 criteria, 18 had HbA1c ≥40 mmol/mol (5.8%). Based on ADA/WHO 2013 criteria, 40 patients were classified with GDM. Ten of these had HbA1c ≥40 mmol/mol (5.8%). Of the 26 pregnant women diagnosed as being positive for GDM by both WHO 1999 and ADA/WHO 2013 criteria, 5 had HbA1c ≥40 mmol/mol (5.8%). The proportion of women classified as positive and negative for the condition by OGTT results and the HbA1c cut-off adopted in this study differed significantly (p <0.001).

## Discussion

In this study, we evaluated the performance of HbA1c test to detect GDM in comparison to a traditional OGTT. Our data showed, as expected, that HbA1c values in pregnant women without GDM were significantly lower than those found in pregnant women with GDM. However, there was some overlap between the HbA1c values displayed by participants in the two groups. These findings are in agreement with other studies, which found mean HbA1c values of 30 to 37 mmol/mol (4.9 to 5.5%) in pregnant women without GDM [22–26]. The presence of anaemia can cause a reduction in HbA1c values, and some studies suggest that this may be one of the reasons for the lower HbA1c levels observed during pregnancy [24]. In our study, anaemia could not explain these differences, since women with and without GDM presented with similar levels of total haemoglobin, within the normal range. We believe that such differences in HbA1c values are more likely to be caused by other physiological processes which take place during pregnancy [25].

The ROC curve analysis used to evaluate the performance of HbA1c test in diagnosing GDM displayed similar AUC values for the WHO 1999 and ADA/WHO 2013 reference criteria (0.714 and 0.756, respectively), indicating an absence of differences in HbA1c performance against the OGTT, regardless of the criteria used to diagnose GDM.

The HbA1c cut-off points of 35 mmol/mol (5.3%) and 36 mmol / mol (5.4%) presented the best equilibrium between sensitivity and specificity when WHO 1999 and ADA/WHO 2013 criteria were used, respectively. However, the sensitivity values for these cut-points were not high enough to allow ruling out GDM diagnoses (68% and 70% for the WHO 1999 and ADA/WHO 2013 criteria, respectively). On the other hand, the use of HbA1c ≥40 mmol/mol (5.8%) as a cut-point presented excellent specificity in ruling in GDM (93% and 95% for WHO 1999 and ADA/WHO 2013 criteria, respectively). A previous study suggested the use of HbA1c ≥42 mmol/mol (5.95%) as a cut-off point to confirm the diagnosis of GDM in women in India (28.6% and 97.2% for sensitivity and specificity, respectively) [24].

When we used the HbA1c ≥40 mmol/mol (5.8%) cut-off point to detect participants with and without GDM, it was found that those classified as having the condition were more likely to be older and to have had previous GDM and a family history of DM, as well as higher BMI, blood pressure (systolic and diastolic), glycemia (fasting, 1h and 2hG) and cholesterol levels. These characteristics have been found to be related to an increased probability of adverse outcomes for both the mother and the babies [2, 3].

As has been previously reported in diagnostic studies of type 2 DM [11–13], the HbA1c test seems to identify a different GDM group from that diagnosed by glucose-based tests. This fact is corroborated by the poor diagnostic agreement between tests.

Our results showed that 38% of GDM cases were diagnosed by the cut-off point of HbA1c ≥40 mmol/mol (5.8%), and that 5% of pregnant women classified as GDM-negative by the OGTT would have been diagnosed as having the condition according to the HbA1c test. The use of the HbA1c test as a diagnostic criterion in this group would identify 9 women with GDM who would not have been identified by the OGTT alone. In agreement with our results, a recent Australian study showed that a subgroup of pregnant women have a normal OGTT but elevated HbA1c, suggesting that HbA1c >40 mmol/mol (5.8%) during pregnancy is a clinically relevant finding [27].

The positive and negative likelihood ratios suggested that a woman with GDM is about five times more likely to have HbA1c levels ≥40 mmol/mol (5.8%) than a woman without the condition. On the other hand, a GDM-negative woman is about three times more likely to have HbA1c concentrations ≤31 mmol/mol (5.0%) than a woman with the disease. The LR+ and LR–were very significant for both HbA1c cut-off points analysed [19,28]. Also, by using Bayes theorem rationale, the post-test probability for GDM was 3.4% for the HbA1c ≤31 mmol/mol (5.0%) cut-point, which is much lower than the pre-test probability of 10%, and 40% for HbA1c ≥40mmol/mol (5.8%), showing a four-fold increase from the pre-test probability [20,28].

The present study did have a few limitations. Firstly, it was a cross-sectional study and the relationship between HbA1c and maternal and/or fetal adverse outcomes could not be analysed. Secondly, our sample of Brazilian pregnant women may differ from other populations, although our results are in accordance with those of other studies which involved different samples [22,26,27]. Third, some limitations are associated with the HbA1c test itself, as its applicability will depend on the observance of all factors affecting HbA1c results. Finally, we did not carry out any cost effectiveness analyses, and the HbA1c is known to be more expensive than glucose-based tests. However, it is possible that the advantages of the HbA1c test may outweigh its disadvantages.

Clinicians should be aware that all diagnostic tests have some limitations and may not always be effective. Although the HbA1c test does not have sufficient sensitivity and specificity to be used as the only diagnostic test for GDM, the use of different HbA1c cut-off points in combination with the OGTT could be useful in detecting the condition, as has also been suggested by other studies in the literature [26, 29]. HbA1c test has several advantages over OGTT, including less biological variation, higher reproducibility, better sample stability and no need of fasting.

In conclusion, different HbA1c cut-off points, in combination with OGTT, may be a useful diagnostic tool for GDM.
